# Chronic jet lag-like conditions dysregulate molecular profiles of neurological disorders in nucleus accumbens and prefrontal cortex

**DOI:** 10.3389/fninf.2022.1031448

**Published:** 2022-12-13

**Authors:** Rabeea Siddique, Faryal Mehwish Awan, Ghulam Nabi, Suliman Khan, Mengzhou Xue

**Affiliations:** ^1^Department of Cerebrovascular Diseases, The Second Affiliated Hospital of Zhengzhou University, Zhengzhou, China; ^2^Henan Medical Key Laboratory of Translational Cerebrovascular Diseases, Zhengzhou, Henan, China; ^3^Department of Medical Lab Technology, The University of Haripur, Haripur, Pakistan; ^4^Institute of Nature Conservation, Polish Academy of Sciences, Kraków, Poland

**Keywords:** circadian rhythms, chronic jetlag (shiftwork), neurological diseases, serotonin, alternating light-dark cycles

## Abstract

**Background:**

Patients with neurological disorders often display altered circadian rhythms. The disrupted circadian rhythms through chronic jetlag or shiftwork are thought to increase the risk and severity of human disease including, cancer, psychiatric, and related brain diseases.

**Results:**

In this study, we investigated the impact of shiftwork or chronic jetlag (CJL) like conditions on mice’s brain. Transcriptome profiling based on RNA sequencing revealed that genes associated with serious neurological disorders were differentially expressed in the nucleus accumbens (NAc) and prefrontal cortex (PFC). According to the quantitative PCR (qPCR) analysis, several key regulatory genes associated with neurological disorders were significantly altered in the NAc, PFC, hypothalamus, hippocampus, and striatum. Serotonin levels and the expression levels of serotonin transporters and receptors were significantly altered in mice treated with CJL.

**Conclusion:**

Overall, these results indicate that CJL may increase the risk of neurological disorders by disrupting the key regulatory genes, biological functions, serotonin, and corticosterone. These molecular linkages can further be studied to investigate the mechanism underlying CJL or shiftwork-mediated neurological disorders in order to develop treatment strategies.

## Introduction

Circadian rhythm disruptions are a major hallmark of neurological and mood disorders (Vadnie and McClung, 2017). The timing of rhythms associated with the central molecular clock and peripheral clocks can be altered due to environmental influences, such as molecular rhythms in mice change with light/dark cycle alterations and restricted food availability (McClung, 2007). These environmental stimuli-mediated changes may be linked with a number of molecular changes (genetic alterations), which can increase the risks of different neurological abnormalities. In humans, the effect of having a desynchronized clock can be experienced by jet lag, shiftwork, changes in seasons, and deprived sleep (McClung, 2007; Woelders et al., 2017). Thus, chronic jetlag or shiftwork can lead to profound mood-related changes in vulnerable individuals (Taylor et al., 1997) including major depressive disorder and bipolar disorder, that share the symptoms including abnormal sleep/wake, appetite, and social rhythms (Dmitrzak-Weglarz et al., 2015). Moreover, the disruption in the normal organization of clock genes can induce the risks of neurological disorders, including major depression disorder (MDD), Schizophrenia, Parkinson’s disease (PD), Alzheimer’s disease (AD), Prader–Willi syndrome (PWS), Smith–Magenis syndrome (SMS), autism spectrum disorders (ASDs), and attention-deficit hyperactivity disorder (ADHD) (Khan et al., 2018; Logan and McClung, 2019). These neurological conditions have widely been reported to be associated with hormonal disregulations (Vincent and Jacobson, 2014). Since disrupted circadian rhythms can affect the functions of serotonin and melatonin, which have been largely known for their critical roles in neurological abnormalities such as depression and bipolar disorder, obsessive-compulsive disorder (McClung, 2007), therefore, investigating these hormones might be helpful in determining the underlying mechanism associated with CJL mediated neurological disorders.

The prominent brain regions “nucleus accumbens, prefrontal cortex, striatum, and hippocampus” are largely involved in several neurological abnormalities. These regions regulate and control neurological functions and physiological activities (Li et al., 2013; Swanson et al., 2017; Khan et al., 2021). In neurological disorders such as Huntington’s disease, Down syndrome and epilepsy, cell-type specific alterations or genetic alterations lead to functional imbalance in brain regions especially striatum, prefrontal cortex and hippocampus (Ghiglieri et al., 2011). Prefrontal cortex and nucleus accumbens are also involved in emotional, cognitive control, and regulation of reward-seeking behaviors (Xue et al., 2022). CJL may increase the risk of neurological diseases and alter the expression levels of genes associated with these diseases in aforementioned brain regions.

Wide range of investigations have focused on finding associations between shiftowrk/chornic (disrupted circadian rhythms) jetlag and diseaes (including cancer and neurological disorders) (Haus and Smolensky, 2013; Li et al., 2013; Van Dycke et al., 2015; Khan et al., 2018; Masri and Sassone-Corsi, 2018). Researchers have determined a link between shiftwork and cancer as indicated by preclinical data support (Filipski et al., 2005; Siegel et al., 2017; Khan et al., 2019), however, such link between shiftwork/chronic jetlag and neurological disorders needs to be developed. Moreover, the molecular mechanism underlying this relationship requires more investigations. Although epidemiological and earlier clinical studies have indicated that disrupted circadian rhythms are connected with brain disorders (Li et al., 2013; Nakazato et al., 2017). However, limited information available that connects shiftwork/chronic jetlag with neurological disorders/diseases-related genes in main regions of the brain. Therefore, we have focused on identification of different genes involved in CJL mediated abnormalities in the brain. In this study, mice were treated with CJL, and then glucose uptake levels, genetics alterations, and hormonal changes were investigated. Insights revealed in this study may help to understand the pathological mechanism underlying neurological disorders induced by CJL and provide a theoretical basis for advances in disease mechanisms, treatment, and prevention.

## Materials and methods

### Animals and chronic jetlag treatment

Both male and female (C57/BL6) mice were obtained from the Model Animal Research Center of Nanjing University. After breeding and obtaining enough numbers, we selected male mice and housed them in standard cages in groups (*N* = 4/5/6). The ambient temperature was 25 ± 1°C, with food and water available *ad libitum*. Mice remained group-housed throughout the experiment, except where single housing was necessary for experiments. Mice were maintained in light-tight housing cabinets. After acclimatization for 1 week under 12 h light: 12 h dark (LD), mice were assigned randomly to regular LD or CJL conditions. The CJL group was exposed to 6 h phase advance every 2 days for a period of 21 days, whereas the lights on and off times were unchanged for the control group throughout the experimental period. For each subsequent experiment, at least three CJL-treated mice, and three control mice were tested and/or dissected at each time point. After 3 days of the last phase advancement at day 21, both CJL treated and control mice were euthanized at ZT1, ZT7, ZT13, and ZT19. After analyzing the running behavior for a 1-week wheel-exposure protocol, animals that that took more than 400 s to start running or that did not reach at least 100 revolutions on the first day of wheel exposure were considered outliers. These animals represented 2 standard deviations away from the group mean were excluded.

### Wheel running activity

Mice were singularly caged and provided with an in-cage running wheel. All mice were given free access to running wheels over a 1-week acclimation period to determine running characteristics of each mouse and to ensure that our randomization was effective in terms of running time and distance. We determined during the acclimatization period that approximately only one percent of wheel activity occurred during the daytime (lights-on).

### Small-animal positron emission tomography scanning

Prior to positron emission tomography (PET) imaging, mice were fasted for 12 h and injected with (250 ± 10 μCi) 18-fluoro-6-deoxy-glucose (FDG) intraperitoneally. After 60 min, mice were anesthetized with 2% isoflurane and images were obtained with the static scanning pattern (10 min) by the Trans-PET Bio-Calibure 700 system (Raycan Technology Co., Ltd, Suzhou, China). The PET images were reconstructed using the three-dimensional (3D) OSEM method with a voxel size of 0.5 × 0.5 × 0.5 mm^3^. A volume-of-interest (VOI) analysis was conducted using the AMIDE software package (The Free Software Foundation Inc., Boston, Massachusetts, USA).

### Brain dissection

Tissues were dissected and snap frozen at four time-points spaced equally through the 24 h LD cycles. The brains of mice were dissected by following the previously published protocol by Spijker (2011). In brief, after cervical dislocation, the head was removed by using surgical scissors to cut from the posterior side of the ears. A midline incision was made in the skin through scissors. The skin was flipped over the eyes to free the skull. Starting from the caudal part at the parietal bone on the top of the skull, a small incision was made with care to avoid damaging the brain. Then a cut was made through the most anterior part of the skull or frontal bone. Hence the brain was removed. The parietal bones at both sides were broken by using curved narrow pattern forceps. In the case where frontal bone remained, a small incision was made to enable breaking and tilting off the bone plate. This was performed with care so that the rupturing of the brain was avoided. After freeing the brain from meninges, it was gently tilted upward by using curved narrow pattern forceps. Then the optic and other cranial nerves were cut by sliding the forceps further down and brain was lifted gently out of the skull. The brain was immediately transferred to metal plate placed on ice to cool down the brain. Excess blood was wiped off.

### Dissection of hippocampus

The brain was placed with the ventral side facing the metal plate. Closed small curved forceps were placed between the cerebral halves and the brain was held with large curved forceps. The forceps were gently opened to open the cortical halves. This step was repeated until the complete opening of the regions. After opening around 60% along the midline, the left cortex was opened from the hippocampus by repeatedly opening the forceps in closed position 30–40° counterclockwise. The same process was repeated for the right cortex by pointing the forceps in a clockwise direction. This movement of forceps was repeated on either side until the hippocampus was visible. Cortex was picked up using the large forceps. Small forceps were used to free the hippocampus from the cortex. The opening and closing process of small forceps was repeated while moving them to the caudal part of the hippocampus/cortex boundary. Possible remainders of cortex were removed by snapping them off using the small forceps. The right and left cortex were removed from the hippocampus in the same way. Small forceps were used to separate the hippocampus from the fornix, hence, two halves of the hippocampus were separated. Hippocampal halves were pushed with closed forceps to the side while keeping the brain in position with the larger forceps. Then hippocampus was removed out of the brain by using the small forceps. The hippocampus was then inspected for the remaining pieces of the cortex and further isolated. The hippocampus was immediately transferred into liquid nitrogen and then stored at −80°C until further process.

### Dissection of prefrontal cortex and striatum

After removal of the hippocampus, the brain was then placed with the dorsal side facing the plate followed by coronal sections approximately 1.0 mm using a sharp and clean blade. Anterior commissure became visible after cutting the olfactory bulb. The first section contains motor cortex, while the subsequent section contains the anterior corpus callosum with a darker area in the middle medial prefrontal cortex (mPFC). After cutting the section containing the mPFC the genus corpus callosum becomes visible. The next section containing capsula externa was cut and followed by a section containing ventral striatum and the last section contained dorsal striatum. Striatum was separated with proper care following the mouse brain atlas and previously published report (Spijker, 2011).

For the mPFC, I took first and second section among. The mPFC, containing the prelimbic and infralimbic cortex, became visible as a darker area between the anterior forceps of corpus callosum and genus corpus callosum. I cut the section through the genus corpus callosum to dissect the mPFC in a diamond-like shape with care to avoid taking any material from the anterior forceps of corpus callosum along. For the striatum, I took the second and third sections. Among these sections, ventral striatum was visible as a darker structure surrounded by the somewhat lighter and less translucent cortex, as well as the anterior forceps of the corpus callosum. In the next section, both the dorsal and ventral striatum appeared darker than the surrounding cortex. I dissected the striatum from genus corpus callosum and adjacent capsula externa, as well as from the ventricle and septum (medial), and cortex (ventral), considering the natural borders. In this last section, I then dissected the dorsal striatum from the corpus callosum and adjacent capsula externa. The dissected mPFC and striatum were immediately transferred to liquid nitrogen then stored at −80°C until further process.

### Dissection of hypothalamus

Removal of the hypothalamus was conducted from the ventral side of the brain. Initially, the optic chiasm was used to locate the hypothalamus. In order to reach the target hypothalamus area, I started cutting off sections (2–3 mm) of the hemispheres using a sharp and clean blade. Then I cut off a thin section near the hypothalamus when the optic chiasm becomes larger and the anterior commissure becomes smaller. Optic chiasm was dissected away from the anterior portion of the hypothalamus, followed by dissection of the mammillary nuclei from the posterior of the hypothalamus. The entire hypothalamus including the arcuate, ventromedial, dorsomedial, and paraventricular nuclei was scooped out using curved forceps. The hypothalamus was immediately transferred into liquid nitrogen and then stored at −80°C until further process.

### Dissection of raphe nuclei

Raphe nuclei were dissected by following the description available in mouse brain Atlas. Two main sections (approximately 1.5–2.0 mm) were cut off from the brain and then a comparatively thinner section approximately 1.0 mm was cut off. The midbrain was exposed by cutting off the upper gray matter regions. Further trimming using a sharp blade exposed the lateral subregions and raphe nucleus. The raphe nucleus was scooped out gently using the forceps. The extra remedial brain regions were trimmed off through the blade. The intact raphe nucleus was immediately transferred into liquid nitrogen and stored in −80°C until further process.

### Dissection of nucleus accumbens

Nucleus accumbens (NA) was dissected by following the previously published protocol with necessary modifications (Matthews et al., 2004). In brief, the brain was placed at an angle such that the olfactory tubercle was leveled in position to produce the desired section. The whole brain was trimmed to expose an angled parasagittal surface and cut sharp blade to yield approximately 1 mm thick brain slice that contained most of the ipsilateral fornix and the central portion of the NA. The NAc was dissected out with care to avoid cutting out the other brain parts. The isolated NAc was further cleaned from any remedial part of other brain and immediately transferred to liquid nitrogen.

### RNA extraction

Total RNA was extracted using TRIzol™ reagent (Thermo Fisher Scientific, China) following the standard protocol provided by the manufacturer. A 1 ml of TRIzol™ reagent per 50–100 mg of tissue was added, and the samples were homogenized using a hand-held homogenizer. The RNA concentration was determined using a spectrophotometer by the following formula;


$$100=\frac{R\,N\,A\,s\,o\,l\,u\,t\,i\,o\,n\,i\,n\,t\,h\,e\,t\,u\,b\,e*c\,o\,n\,c.o\,f\,n\,a\,n\,o\,d\,r\,o\,p}{R\,N\,A\,s\,o\,l\,u\,t\,i\,o\,n\,i\,n\,t\,h\,e\,t\,u\,b\,e+X}$$

### Primers design

Primers were designed using PRIMER BLAST, PRIMER-3, and PRIMER-5 (Supplementary Table 1). The average melting temperature was selected as 60°C, and the PCR product size was 70–200 bp. The average primer size selected was 20 bp, whereas the average *GC* content (%) was 50%. All the primers were purchased from AuGCT DNA-SYN Biotechnology, China.

### Quantitative PCR

cDNA was synthesized using high capacity RNA to cDNA kit (Transgen, Biotech, China). qPCR was performed using Trans Master Mix (Transgen, Biotech, China) and an applied biosystems 96 well thermal cycler (Thermo Fisher Scientific). The master mix was prepared according to the manufacturer’s protocol with certain modifications according to the requirements. For each reaction, 10 μl qPCR mix (2X), 0.4 μl forward primer (1 nmol), 0.4 μl of reverse primer (1 nmol), and 0.4 μl reference dye were used per reaction. Furthermore, 1 or 1.4 μl of cDNA or RNA (200 or 100 ng/μl) was added to each reaction. Fold changes were normalized to GAPDH endogenous reference gene, and calculation was done using the ΔΔCt method (Livak and Schmittgen, 2001).

### Corticosterone measurement

A corticosterone ELISA kit (96 wells) was purchased from Enzo life sciences (United States). Corticosterone was extracted from the blood according to the protocol provided by the kit’s manufacturer. Briefly, all the solutions were brought to room temperature. Ten microliters of each sample was put into a microfuge tube. One milliliter 1:100 Steroid Displacement Reagent (SDR) solution was diluted in deionized water immediately before use. Ten microliters diluted SDR was added to each sample tube. Tubes were briefly vortexed and incubated for approximately 5 min before diluting with EIA buffer. Then 380 μl EIA assay buffer was added to each tube and vortexed. The solutions were further diluted up to 1:140, and the concentration was measured through an ELISA plate reader (FlexStation 3, San Jose, CA 95134, USA). The actual concentration was measured by comparing the samples with standard solutions. Corticosterone levels were measured at four time points after treating the animals with 6 h phase advance for 4 weeks. Mice were treated with 6 h phase advance for 4 weeks. Blood was collected from tails at four different time points. Mice were handled carefully to avoid stress. For each time point experiment, three mice were used from each group.

### Serotonin (5-HT) measurements

After sacrificing the mouse, the brains were immediately removed and kept at −80^°^C. Different brain regions were then dissected. After weighing, specimens were homogenized in (1.4 ml per 50 mg tissue) of acid butyl alcohol and centrifuged at 3,000 g for 5 min before collecting the supernatant. Then 2.5 ml n-heptane and 0.5 ml 0.1 N HCl were added to the supernatant, and the mixture was vortexed for 5 min followed by centrifugation at 3,000 g for 5 min at room temperature. The water phase (0.25 ml) was collected and mixed with 0.05 ml of 0.5% cysteine and 1.25 ml of 0.006% o-phenyl-di-formaldehyde in 10 N HCl. After mixing thoroughly, the solution was placed into boiling water for 10 min. After the solution was cooled to room temperature in water, the fluorescence intensity of 5-HT was measured at 365/480 nm by a fluorescence spectrophotometer (PerkinElmer, USA). The 5-HT concentration was determined by comparing it to a standard 5-HT dilution series.

### RNA sequencing analysis and library preparation process

The prefrontal cortex and nucleus accumbens tissues were dissected from the mouse brain at zeitgeber time (ZT)1 and ZT13 and stored at −80°C. Total RNA was extracted and RNA sequencing was conducted (Vazyme Biotech, Nanjing China).

### RNA quality assessment

RNA purity was detected by NanoDrop^®^ spectrophotometers (Thermo Fishser, MA, USA). The RNA concentration was measured using Qubit^®^ RNA Assay Kit in Qubit^®^ 3.0 Fluorometer (Life Technologies, CA, USA). RNA integrity was assessed using the RNA Nano 6000 Assay Kit of the Bioanalyzer 2100 system (Agilent Technologies, CA, USA).

### Library preparation for mRNA-seq

A total amount of 1 μg qualified RNA per sample was used as input material for the library preparation. The sequencing libraries were generated using the VAHTS mRNA-seq v2 Library Prep Kit for Illumina^®^ (Vazyme, NR601) following the manufacturer’s recommendations. Firstly, mRNA was purified from total RNA using poly-T oligo-attached magnetic beads. Fragmentation was performed using divalent cations under elevated temperature in Vazyme Frag/Prime Buffer. The cleaved RNA fragments were copied into first-strand cDNA using reverse transcriptase and random primers. Second strand cDNA synthesis was subsequently performed using buffer, dNTPs, DNA polymerase I, and RNase H. Then, the cDNA fragments were end-repaired with adding a single “A” base at the 3’-end of each strand, ligated with the special sequencing adapters (Vazyme, N803) subsequently. The products were purified, and the size was selected with VAHTSTM DNA Clean Beads (Vazyme, N411) to get the appropriate size for sequencing. Finally, PCR was performed and aimed products were purified.

### Library examination

Library concentration was initially measured using Qubit^®^ RNA Assay Kit in Qubit^®^ 3.0. Insert size was assessed using the Agilent Bioanalyzer 2100 system. After the insert size was deemed consistent with expectations, it was accurately quantified using qPCR by Step One Plus Real-Time PCR system (ABI, USA).

### Library clustering and sequencing

The clustering of the index-coded samples was performed on a cBot Cluster Generation System (Illumina, USA) according to the manufacturer’s instructions. After cluster generation, the library preparations were sequenced on an Illumina Hiseq X Ten platform and 150 bp paired-end module.

### Data processing and analysis

The raw reads were filtered to produce clean data by excluding sequencing adaptors, and low-quality reads, including reads containing over 5% “N” and those containing >50% bases with a quality value less than 10. The quality assessment of clean data was performed using FastQC v0.11.9. Sequencing reads were then aligned to the mouse mm10 reference genome using HISAT2. The DESeq2 performed the differential expression analysis with default parameters. The significance threshold of *P* < 0.05 was applied. The number of differentially expressed genes (DEGs) and the number of genes that exhibited a fold-change >2 in each brain tissue at each time point were displayed in Table 1. DAVID online tool was used for Gene Ontology and KEGG pathway analysis.

**TABLE 1:** Psychiatric disorders associated genes were differentially expressed under CJL.

ZT/Region	Ensemble ID	Gene symbol	Gene name	Fold change	*P*-value	Diseases/Disorder	GO term	Related pathways
ZT13/NA	ENSMUSG00000 004668	Abca13	ATP binding cassette subfamily A member 13	5.78	0.005901174	Shwachman-diamond syndrome 1 and schizophrenia	ATPase and cholesterol transporter activity	CDK-mediated phosphorylation and removal of Cdc6 and innate immune system
ZT13/NA	ENSMUSG00000 031654	Cbln1	Cerebellin 1 precursor	4.32	0.001178095	Depression	Protein homodimerization	
ZT13/NA	ENSMUSG00000 022935	Grik1	Glutamate ionotropic receptor kainate type subunit 1	1.328	0.00669943	Monosomy 21 and nondisjunction.	Onotropic glutamate receptor activity and	Transmission across chemical synapses and presynaptic function of kainate receptors
ZT13/NA	ENSMUSG0000 0085830	Grin1os	Glutamate receptor, ionotropic	0.72	0.005276759			
ZT13/NA	ENSMUSG00000 002771	Grin2d	Glutamate ionotropic receptor	1.40	0.020291805			
ZT13/NA	ENSMUSG00000 023192	Grm2	Glutamate metabotropic receptor 2	2.08	0.00019965	Schizophrenia and amphetamine abuse	G-protein coupled receptor activity and glutamate receptor activity	Peptide ligand-binding receptors and signaling by GPCR
ZT13/NA	ENSMUSG00000 063239	Grm4	Glutamate metabotropic receptor 4	0.73	0.024102966	Epilepsy, idiopathic generalized 10 and schizophrenia	G-protein coupled receptor activity and calcium-dependent protein binding	Peptide ligand-binding receptors and Signaling by GPCR
ZT13/NA		Grm8	Glutamate metabotropic receptor 8	1.71	0.0000544	Schizophrenia	GPCRs	G-protein coupled receptor activity and group III metabotropic glutamate receptor activity.
ZT13/NA	ENSMUSG00000 032269	Htr3a	5-Hydroxytryptamine receptor 3A	1.64	0.010275407	Serotonin syndrome and motion sickness.	Extracellular ligand-gated ion channel activity	Sudden infant death syndrome (SIDS) susceptibility pathways and transport of glucose and other sugars.
ZT13/NA	ENSMUSG0000 0028747	Htr6	5-Hydroxytryptamine receptor 6	0.71	0.037223485	Schizophrenia 5 and acute stress disorder	G-protein coupled receptor activity and histamine receptor activity	Monoamine GPCRs and signaling by GPCR
ZT13/NA	ENSMUSG00000 059857	Ntng1	Netrin G1	1.57	0.028761388	Rett syndrome and schizophrenia		Metabolism of proteins and post-translational modification- synthesis of GPI-anchored proteins
ZT13/NA	ENSMUSG0000 0043811	Rtn4r	Reticulon 4 receptor	1.58	0.039552183	Schizophrenia and acute lymphocytic leukemia		Cytoskeleton remodeling Regulation of actin cytoskeleton by Rho GTPases and Guidance Cues and Growth Cone Motility
ZT13/NA	ENSMUSG0000 0032902	Slc16a1	Solute carrier family 16 member 1	1.26	0.005070062	Erythrocyte lactate transporter defect and hyperinsulinemic hypoglycemia, familial, 7	Protein homodimerization activity and monocarboxylic acid transmembrane transporter activity.	Basigin interactions and metabolism
ZT13/NA	ENSMUSG0000 0032122	Slc37a2	Solute carrier family 37 member 2	0.68	0.046427607	Caffey disease	Transporter activity	
ZT13/NA	ENSMUSG0000 0021565	Slc6a19	Solute carrier family 6 member 19	3.20	0.019754465	Hartnup disorder and iminoglycinuria	Neurotransmitter: sodium symporter activity and neutral amino acid transmembrane transporter activity	Transport of glucose and other sugars, bile salts and organic acids, metal ions and amine compounds and Mineral absorption
ZT13/NA	ENSMUSG0000 0011171	Vipr2	Vasoactive intestinal peptide receptor 2	5.55	0.006572358	Schizophrenia 16 and holoprosencephaly 3.	G-protein coupled receptor activity and vasoactive intestinal polypeptide receptor activity	Sudden infant death syndrome (SIDS) susceptibility pathways and signaling by GPCR.
ZT1/NA	ENSMUSG00000 004668	Abca13	ATP binding cassette subfamily A member 13	5.142	0.010892494	Shwachman-diamond syndrome 1 and schizophrenia	Atpase activity and cholesterol transporter activity	CDK-mediated phosphorylation and removal of Cdc6 and innate immune system
ZT1/NA	ENSMUSG00000 024222	Fkbp5	FK506 binding protein 5	0.725	0.000526409	Major depressive disorder and post-traumatic stress disorder	Peptidyl-prolyl cis-trans isomerase activity and FK506 binding	Glucocorticoid receptor regulatory network and farnesoid X receptor pathway
ZT1/NA	ENSMUSG0000 0029212	Gabrb1	Gamma-aminobutyric acid type A receptor beta1 subunit	1.27	0.04209984	Epileptic encephalopathy, early infantile, 45 and schizoaffective disorder	Chloride channel activity and GABA-A receptor activity	Akt signaling and neurophysiological process glutamate regulation of dopamine D1A receptor signaling
ZT1/NA	ENSMUSG00000 002771	Grin2d	Glutamate ionotropic receptor NMDA type subunit 2D	1.56	0.002340667	Epileptic encephalopathy, early infantile, 46 and undetermined early-onset epileptic encephalopathy	Ionotropic glutamate receptor activity and NMDA glutamate receptor activity	Amyotrophic lateral sclerosis (ALS) and neurophysiological process glutamate regulation of dopamine D1A receptor signaling
ZT1/NA	ENSMUSG0000 0028747	Htr6	5-Hydroxytryptamine receptor 6	0.72	0.04693987	Schizophrenia 5 and acute stress disorder	G-protein coupled receptor activity and histamine receptor activity	Monoamine GPCRs and signaling by GPCR
ZT1/NA	ENSMUSG00000 006522	Itih3	Inter-alpha-trypsin inhibitor heavy chain 3	1.62	0.00164351	Schizophrenia and major depressive disorder	serine-type endopeptidase inhibitor activity and endopeptidase inhibitor activity.	Response to elevated platelet cytosolic Ca2+ and cell adhesion_cell-matrix glycoconjugates
ZT1/NA	ENSMUSG0000 0034755	Pcdh11x	Protocadherin 11 X-linked	1.28	0.03337531	Dyslexia and schizoaffective disorder	calcium ion binding	
ZT1/NA	ENSMUSG0000 0031398	Plxna3	Plexin A3	1.33	0.000730354	Childhood-onset schizophrenia	Semaphorin receptor activity	Developmental biology
ZT1/NA	ENSMUSG0000 0017969	Ptgis	Prostaglandin I2 synthase	0.67	0.013953188	Hypertension, schizophrenia		Adipogenesis and metabolism
ZT1/NA	ENSMUSG0000 0043811	Rtn4r	Reticulon 4 receptor	0.63	0.04001688	Schizophrenia and acute lymphocytic leukemia		Cytoskeleton remodeling Regulation of actin cytoskeleton by Rho GTPases and guidance cues and growth cone motility
ZT1/NA	ENSMUSG0000 0019970	Sgk1	Serum/Glucocorticoid regulated kinase 1	0.045	9.90934E-18	Gastric cancer and Liddle syndrome	Transferase activity, transferring phosphorus-containing groups and protein tyrosine kinase activity	Gene expression and glucocorticoid receptor regulatory network
ZT1/NA	ENSMUSG0000 0018459	Slc13a3	Solute carrier family 13 member 3	0.73	0.008700156	N-acetylglutamate synthase deficiency	Transporter activityand high-affinity sodium: dicarboxylate symporter activity	Transport of glucose and other sugars, bile salts and organic acids, metal ions, and amine compounds
ZT1/NA	ENSMUSG0000 0029843	Slc13a4	Solute carrier family 13 member 4	0.24	3.88864E-05		Include transporter activity and sodium: sulfate symporter activity	Transport of glucose and other sugars, bile salts and organic acids, metal ions and amine compounds
ZT1/NA	ENSMUSG0000 0052912	Smarca5-ps	SWI/SNF related, matrix associated,	3.30	0.01672786			
ZT1/NA	ENSMUSG00000 007944	Ttc9b	Tetratricopeptide Repeat domain 9B	0.73	0.03778283	Endogenous depression	Peptidyl-prolyl cis-trans isomerase activity and FK506 binding	
ZT13/PFC	ENSMUSG0000 0056629	Fkbp2	FK506 binding protein 5	1.31	0.006556677	Major depressive disorder and post-traumatic stress disorder	Peptidyl-prolyl cis-trans isomerase activity and FK506 binding	Glucocorticoid receptor regulatory network and farnesoid X receptor pathway
ZT13/PFC	ENSMUSG0000 0024912	Fosl1	FOS like 1, AP-1 transcription factor subunit	1.31	0.029305593	T-cell leukemia and Pfeiffer syndrome	DNA binding transcription factor activity and RNA polymerase	ERK signaling and integrated breast cancer pathway
ZT13/PFC	ENSMUSG0000 0029212	Gabrb1	Gamma-aminobutyric acid type A receptor beta1 subunit	0.71	0.005182001	Epileptic encephalopathy, early infantile, 45 and schizoaffective disorder	Chloride channel activity and GABA-A receptor activity	Akt signaling and neurophysiological process glutamate regulation of dopamine D1A receptor signaling
ZT13/PFC	ENSMUSG0000 0030209	Grin2b	Glutamate ionotropic receptor NMDA type subunit 2B	0.72	0.002135375	e Mental retardation, autosomal dominant 6, with or without seizures and epileptic encephalopathy, early infantile, 27.	Calcium channel activity and ionotropic glutamate receptor activity	Amyotrophic lateral sclerosis (ALS) and neurophysiological process glutamate regulation of dopamine D1A receptor signaling
ZT13/PFC	ENSMUSG0000 0034997	Htr2a	5-Hydroxytryptamine receptor 2A	0.65	0.018803838	Major depressive disorder and obsessive-compulsive disorder	G-protein coupled receptor activity and drug binding	GPCRs, other and sudden infant death syndrome (SIDS) susceptibility pathways
ZT13/PFC	ENSMUSG0000 0024525	Impa2	Inositol monophosphatase 2	1.46	0.022870352	Bipolar disorder and febrile seizures	Protein homodimerization activity and inositol monophosphate 3-phosphatase activity	Inositol phosphate metabolism and metabolism
ZT13/PFC	ENSMUSG0000 0039057	Myo16	Myosin XVI	1.53	0.035595955	Schizophrenia	Actin binding and actin filament binding	
ZT13/PFC	ENSMUSG0000 0026435	Slc45a3	Solute carrier family 45 member 3	2.14	0.016938843	Prostate cancer and suppression of tumorigenicity 12		MicroRNAs in cancer and metabolism
ZT13/PFC	ENSMUSG0000 0031099	Smarca1	SWI/SNF related, matrix associated	0.69	0.023613024	Rhabdoid tumor predisposition syndrome 1 and Coffin-Siris syndrome 3	Transcription coactivator activity and RNA polymerase II	Transcription ligand-dependent activation of the ESR1/SP pathway and PEDF induced signaling
ZT13/PFC	ENSMUSG0000 0031715	Smarca5	SWI/SNF related, matrix associated	0.72	0.012084672	Extraskeletal Ewing sarcoma and Williams-Beuren syndrome	Nucleic acid binding and hydrolase activity	Cell cycle, mitotic and activated PKN1 stimulates transcription
ZT13/PFC	ENSMUSG0000 0029920	Smarcad1	SWI/SNF-related, matrix-associated	0.74	0.019590473	Adermatoglyphia and Basan syndrome	Nucleic acid binding and helicase activity	Transcriptional regulatory network in embryonic stem cell and PEDF induced signaling.
ZT1/PFC	ENSMUSG0000 0063889	Crem	CAMP responsive element modulator	1.37	0.001832659	Female stress incontinence and type 1 diabetes mellitus 10	DNA binding transcription factor activity and core promoter	Transcription_CREM signaling in testis and sudden infant death syndrome (SIDS) susceptibility pathways
ZT1/PFC	ENSMUSG0000 0024222	Fkbp5	FK506 binding protein 5	0.74	0.001751921	Major depressive disorder and post-traumatic stress disorder	Peptidyl-prolyl cis-trans isomerase activity and FK506 binding.	Glucocorticoid receptor regulatory network and farnesoid X receptor pathway
ZT1/PFC	ENSMUSG00000 003974	Grm3	Glutamate metabotropic receptor 3	1.26	0.023136716	Schizophrenia and bipolar disorder	G-protein coupled receptor activity and glutamate receptor activity	Peptide ligand-binding receptors and signaling by GPCR
ZT1/PFC	ENSMUSG00000 002930	Ppp1r17	Protein phosphatase 1 regulatory subunit 17	19.35	0.006708572	Hypercholesterolemia, familial	Protein phosphatase inhibitor activity and phosphatase inhibitor activity.	Long-term depression
ZT1/PFC	ENSMUSG0000 0032902	Slc16a1	Solute carrier family 16 member 1	1.39	5.60393E-05	Erythrocyte lactate transporter defect and hyperinsulinemic hypoglycemia, familial, 7	Protein homodimerization activity and monocarboxylic acid transmembrane transporter activity	Basigin interactions and metabolism
ZT1/PFC	ENSMUSG0000 0020100	Slc29a3	Solute carrier family 29 member 3	1.25	0.003517058	Histiocytosis-lymphadenopathy plus syndrome	Nucleoside transmembrane transporter activity	Transport of glucose and other sugars, bile salts and organic acids, metal ion, vitamins and nucleosides.
ZT1/PFC	ENSMUSG0000 0019970	Sgk1	Serum/Glucocorticoid regulated kinase 1	0.63	1.10114E-06	Gastric cancer	Transferase and protein tyrosine kinase activity	Gene expression

NA, nucleus accumbens; PFC, prefrontal cortex.

### Bioinformatics analysis

The DAVID online tool was used for Gene Ontology, KEGG pathway analysis, and other related bioinformatics analyses.

### Sleep analysis using the piezoelectric system

Piezo-Sleep System is a piezoelectric sensor system comprised of Plexiglas cages lined with piezoelectric films across the entire cage floor (Signal Solutions LLC, Lexington, KY, United States). The highly sensitive piezoelectric films sense the pressure changes caused by movements. Wakefulness, in terms of active and resting, is characterized in response to weight shifting and voluntary body movements, which are detected by irregular transient and high amplitude pressure variations whereas sleep is characterized by quasi-periodic signals with low variations in amplitude. Percent sleep/wake statistics were calculated by sleep-wake decisions in the 2-s intervals which were binned over specified time periods. Mean sleep bout lengths were computed using the durations of uninterrupted runs of sleep state labels. The Bout length count is initiated when a 30-s interval contains more than 50% sleep and terminates when a 30-s interval has less than 50% sleep to eliminate the impact of short and ambiguous arousals on the bout length statistic. Sleep was analyzed for day 11, day 13, day 31, and day 33 using the analysis software and manual provided by the manufacturer.

### Statistical analysis

All results presented were analyzed using Excel (Microsoft Software, 365) and GraphPad Prism 7 (GraphPad Software, La Jolla, California, USA). Student’s *t*-test and one-way and two-way ANOVA have been performed accordingly. Statistical significance was determined using the Holm-Sidak method, with alpha = 0.05. Each row was analyzed individually, without assuming a consistent SD.

### Availability of data and materials

Sequencing data and the list of differentially expressed genes can be accessed at NCBI Gene Expression Omnibus (GEO) with the accession number GSE153540.^1^ Data related to PET analysis and imaging can be found at figshare.^2^ Further data analyzed for the genes associated with neurological diseases can be obtained from the corresponding author on reasonable request.

## Results

### Chronic jetlag altered a number of genes and pathways linked with neurological diseases in prefrontal cortex and nucleus accumbens

**FIGURE 1:**
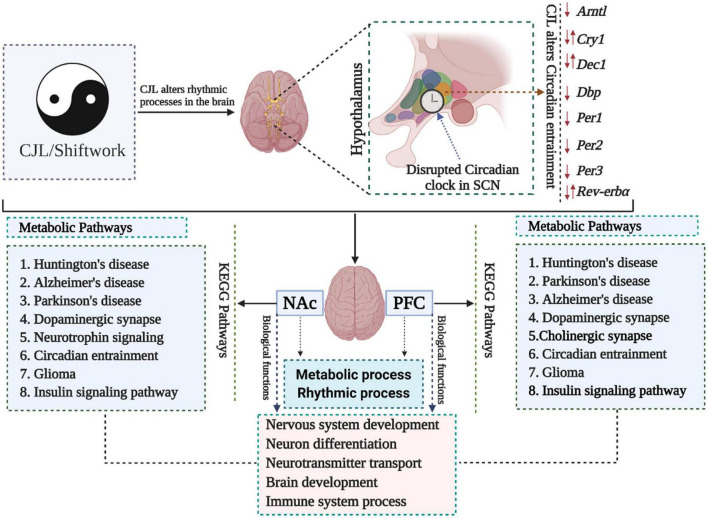
Chronic jetlag (CJL) mediated molecular alterations in the brain. Expression levels of a number of circadian clock-related genes were altered in mice exposed to CJL, indicating that the circadian clock was disrupted. In addition, metabolic pathways were also found altered in response to CJL. Moreover, pathways associated with important neurological diseases and biological functions were found affected by CJL.

We measured the transcriptome profile of the prefrontal cortex (PFC) and nucleus accumbens (NAc) (Figure 1 and Supplementary Figures 1, 2; Gao et al., 2020). Kyoto Encyclopedia of Genes and Genomes (KEGG) analysis revealed that Huntington’s diseases, Alzheimer’s disease, Parkinson’s disease, dopaminergic synapse signaling pathway, glioma, and insulin signaling pathway were among the most affected pathways in PFC and NAc (Supplementary Figure 1). Gene Ontology (GO) analysis revealed that CJL altered key biological functions, including nervous system development, neuron differentiation, brain development, neurotransmitter transport, and immune system process.

The mRNA levels of several genes associated with neurological disorders (such as depressive disorders) were found altered in NAc and PFC, as depicted by RNA sequencing analysis. Moreover, some of key regulatory genes including *Smarcad1*, *Drd1*, *Drd2*, *Fosb*, *Grik2*, *Grin2a*, *Grin2b*, *Hdac1*, *Hdac9*, *Htr2a*, *Jarid2*, *Sin3a*, *Sirt2*, *Slc13a5*, *Slc16a1*, *Slc29a1*, *Slc2a1*, *Slc2a3*, *Slc37a4*, *Slc45a1*, and *Smarca1* were measured in NAc, PFC, hippocampus, hypothalamus and striatum using qPCR. These genes were differentially expressed in mice treated with CJL (Table 1).

### Chronic jetlag altered genes associated with depressive disorders

**FIGURE 2:**
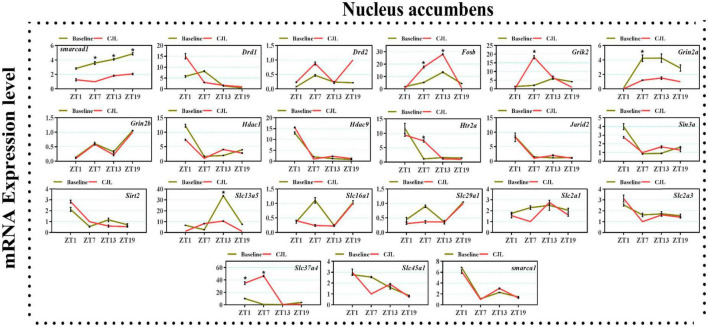
Chronic jetlag (CJL) altered mRNA levels of genes associated with psychiatric disorders in the NAc. This figure shows mRNA levels in NAc extracts from baseline and CJL treated animals (*n* = 3 per group), as assayed by three independent qPCR assays, at four different time points (ZT1, ZT7, ZT13, and ZT19). Expression levels in CJL treated mice were normalized to selected expression levels in baseline mice at specific ZT. Results are expressed as mean ± SEM. *P < 0.01, two-way ANOVA.

**FIGURE 3:**
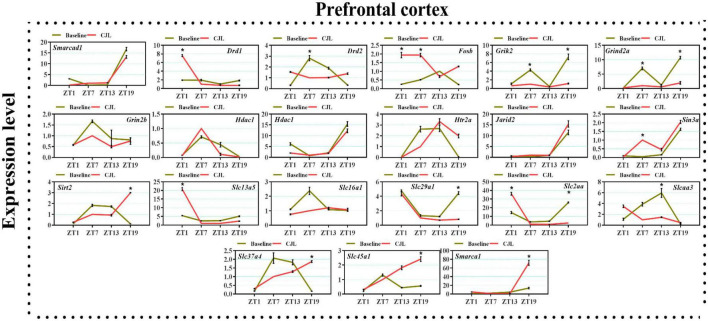
Chronic jetlag (CJL) altered mRNA levels of genes associated with psychiatric disorders in the PFC. This figure shows mRNA levels in PFC extracts from baseline and CJL treated animals (*n* = 3 per group), as assayed by three independent qPCR assays, at four different time points (ZT1, ZT7, ZT13, and ZT19). Results are expressed as mean ± SEM. *P < 0.01, two-way ANOVA.

**FIGURE 4:**
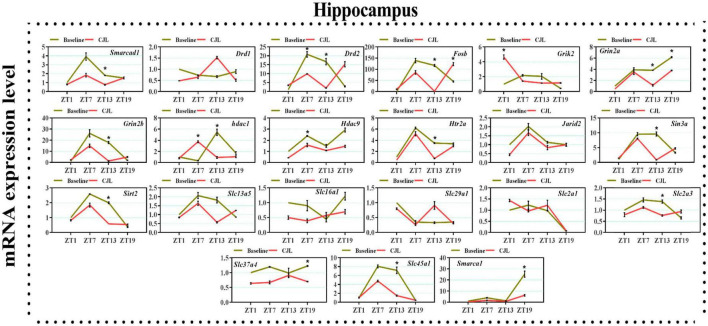
Chronic jetlag (CJL) altered mRNA levels of genes associated with psychiatric disorders in the hippocampus. This figure shows mRNA levels in hippocampus extracts from baseline and CJL treated animals (*n* = 3 per group), as assayed by three independent qPCR assays, at four different time points (ZT1, ZT7, ZT13, and ZT19). Results are expressed as mean ± SEM. *P < 0.01, two-way ANOVA.

**FIGURE 5:**
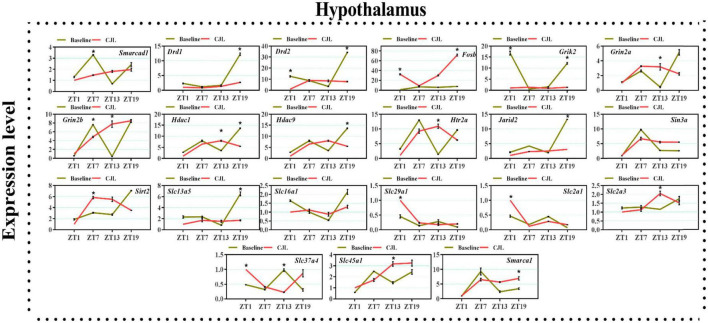
Chronic jetlag (CJL) altered mRNA levels of genes associated with psychiatric disorders in the hypothalamus. This figure shows mRNA levels in hypothalamus extracts from baseline and CJL treated animals (*n* = 3 per group), as assayed by three independent qPCR assays, at four different time points (ZT1, ZT7, ZT13, and ZT19). Results are expressed as mean ± SEM. *P < 0.01, two-way ANOVA.

**FIGURE 6:**
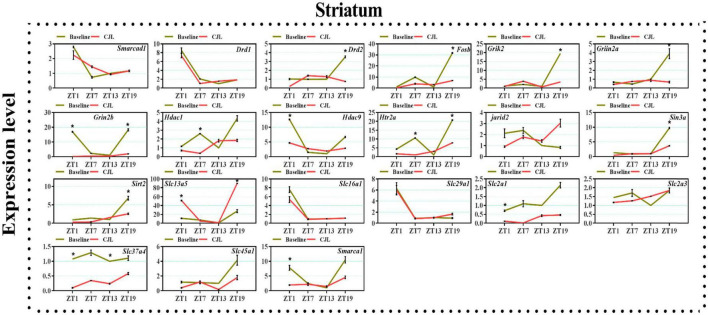
Chronic jetlag (CJL) altered mRNA levels of genes associated with psychiatric disorders in the striatum. This figure shows mRNA levels in striatum extracts from baseline and CJL treated animals (*n* = 3 per group), as assayed by three independent qPCR assays, at four different time points (ZT1, ZT7, ZT13, and ZT19). Results are expressed as mean ± SEM. *P < 0.01, two-way ANOVA.

We found a number of genes associated with psychiatric disorders showed altered expression under CJL (Figures 2–6), including several serotonin receptors *htr2a*, *htr4*, *htr7*, *htr5b*, *htr6*, and *htr3a*. These genes have been found to be involved in depression-related processes (Serretti et al., 2007; Lohoff et al., 2011; Nautiyal and Hen, 2017). We further measured the mRNA levels of a number of genes known to be involved in the pathology of psychiatric disorders, including *Bdnf* (Schmidt et al., 2011), *Fosb* (Heller et al., 2014), *Drd1/2* (Le-Niculescu et al., 2011; Zhang et al., 2014), *Grik2* (Shibata et al., 2002), *Hdac1/9v* (Macpherson et al., 2015; Wu et al., 2015; Subburaju et al., 2016), and *Grin2a/b* (Lemke et al., 2014; Liu et al., 2015). The mRNA levels of several stress and/or depression related genes including Smarcad1, Drd1, Drd2, Fosb, Grik2, Grin2a, Grin2b, Hdac1, Hdac9, Htr2a, Jarid2, Sin3a, Sirt2, Slc13a5, Slc16a1, Slc29a1, Slc2a1, Slc2a3, Slc37a4, Slc45a1, and Smarca1 were measured in NAc, PFC, hippocampus, hypothalamus, and striatum using qPCR (Figures 2–6). In the case of NAc (Figure 2), Fosb, Grik2, Htr2a, and Slc37a4, smarcad1, Grin2a, and Slc13a5 were either upregulated or downregulated at-least at one time-point. In PFC (Figure 2) Drd2, Fosb, Sin3a, Sirt2, Slc13a5, Slc37a4, and Smarca1 were upregulated whereas, Drd2, Grin2a, Slc2a1, and Slc2a3, were downregulated on CJL exposure at-least at one time-point In the hippocampus (Figure 4), only Grik2 was found upregulated at ZT-1, while Smarcad1, Fosb, Drd2, Grin2a, Grin2b, Hdac9, Htr2a, Sin3a, Sirt2, Slc2a3, Slc37a4, Slc45a1, and Smarca1 were downregulated at either one time-point or more than time-points. Similarly, in hypothalamus (Figure 5) Fosb, Gin2a, Htr2a, Sirt2, Slc29a1, Slc2a1, Slc2a3, Slc45a1, and Smarca1 were upregulated whereas Drd1, Grik2, Hdac9, Jarid2, and Slc13a5 were downregulated at-least at one time-point. In the striatum (Figure 6), we found that Slc13a5 was upregulated, whereas Drd2, Fosb, Grik2, Grin2a, Grin2b, Hdac1, Hdac9, Htr2a, Sin3a, Sirt2, Slc2a1, Slc37a4, and Smarca1 were downregulated.

### Chronic jetlag altered clock genes in the hypothalamus and raphe nucleus

Since CJL is known to alter the molecular clock (Bollinger and Schibler, 2014; Swanson et al., 2017; West et al., 2017), we examined mRNA levels of circadian clock genes and found that *Arntl*, *Cry1*, *Dbp*, *Dec1*, *Per1*, *Per2*, *Per3*, and *Rev-erbα* were altered in the hypothalamus of mice exposed to CJL (Supplementary Figures 4, 5). Moreover, we examined mRNA levels of clock genes, *Arntl*, *Cry1*, *Cry2*, *Clock*, *Dbp*, *Dec1*, *Dec2*, *Nr1d1*, *Nr1d2*, *Nfil3*, *Per1*, *Per2*, *Per3*, *Rorα*, *Rorβ*, and *Rorγ* in raphe nucleus (Supplementary Figure 5). Since CJL is known to alter the molecular clock (Bollinger and Schibler, 2014; Swanson et al., 2017; West et al., 2017), I verified this by examining mRNA levels of clock genes, *Arntl*, *Cry1*, *Cry2*, *Clock*, *Dbp*, *Dec1*, *Dec2*, *Nr1d1*, *Nr1d2*, *Nfil3*, *Per1*, *Per2*, *Per3*, *Rorα*, *Rorβ*, and *Rorγ* in raphe nucleus whereas *Arntl*, *Cry1*, *Dbp*, *Dec1*, *Per1*, *Per2*, *Per3*, and *Rev-erbα* in PFC, NAc, hippocampus, hypothalamus, and striatum. We found *Arntl*, *Cry1*, *Dec2*, *Nr1d1*, and *Nfil3* to be significantly altered in the raphe nucleus *Arntl*, *Cry1*, *Dec1*, *Per1*, *Per3*, and *Rev-erbα* in PFC, *Arntl*, *Cry1*, *Dbp*, *Dec1*, *Per1*, *Per2*, *Per3*, and *Rev-erbα* in NAc (Supplementary Figure 5).

### Chronic jetlag affected brain metabolism

Given the effects of CJL on the brain, its activity was assessed by using PET scanning. We observed that the CJL caused a decrease in glucose levels in all regions of the brain. These results imply that brain activation or glucose metabolism is largely affected by CJL (Supplementary Figure 6).

### Chronic jetlag disrupted serotonin regulation in the brain

**FIGURE 7:**
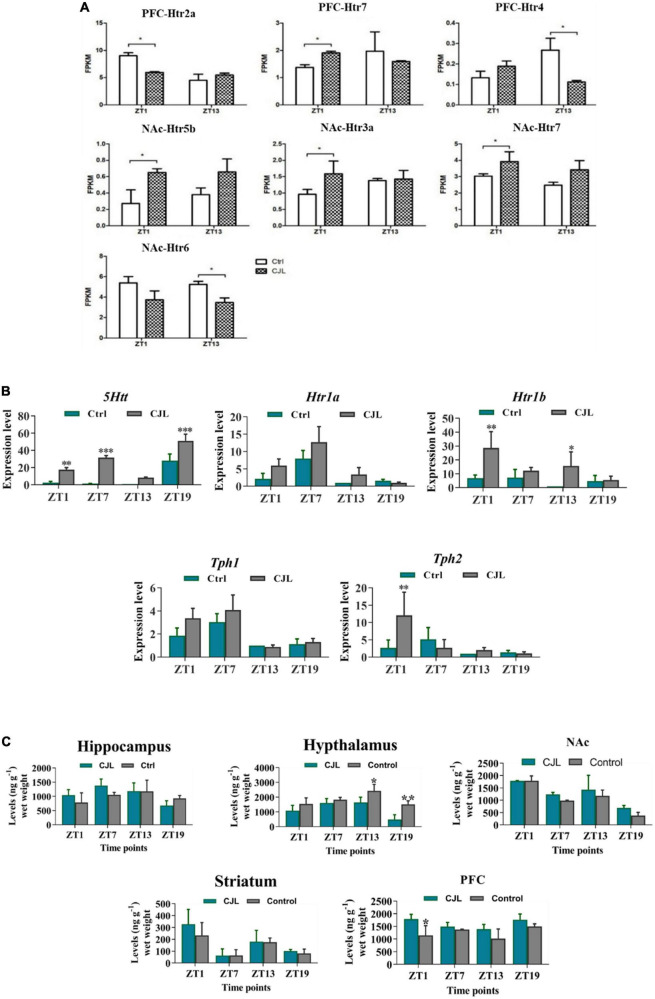
Chronic jetlag (CJL) disrupts serotonin regulation in the brain. **(A)** CJL altered the levels of 5-HT receptors and transporters in the nucleus accumbens and prefrontal cortex (detected by RNA sequencing). All data are presented as means ± SEM, *P < 0.05. **(B)** CJL altered *5Htt* mRNA in the raphe nucleus. This figure shows mRNA levels in raphe nucleus extracts from baseline and CJL treated animals (*n* = 3 per group), as assayed by three independent qPCR assays, at four different time points (ZT1, ZT7, ZT13, and ZT19). Results are expressed as mean ± SEM. **P < 0.01, ***P < 0.001, two-way ANOVA. Panel **(C)** represents levels (mean ng g^– 1^ wet weight ± SEM) of dopamine (5-HT) in the hippocampus, hypothalamus, striatum, frontal cortex, accumbens as determined by fluorospectrophotometry. *P < 0.05, **P < 0.01, ***P < 0.001 (*n* = 304), two-way ANOVA.

RNA sequencing analysis revealed that the CJL affected the expression of serotonin receptors in NAc and PFC (Figure 7). Overall, *htr2a*, *htr4*, *htr7*, *htr5b*, *htr6*, and *htr3a* were altered in mice treated with CJL. To confirm that CJL can affect serotonin regulation, we tested serotonin levels in different brain regions. Our results indicated that the concentration of serotonin was significantly decreased in the hypothalamus and increased in PFC of mice treated with CJL compared to the baseline. Since the raphe nucleus is an important brain region involved in the production and regulation of serotonin, we further examined the mRNA levels of *5htt*, tryptophan hydroxylase (*Tph1/2*), and *Htr1A/1B*. *Htr1B* and *Tph2* mRNA levels were significantly increased in the raphe nucleus in mice after CJL treatment.

### Chronic jetlag altered corticosterone levels in the blood

**FIGURE 8:**
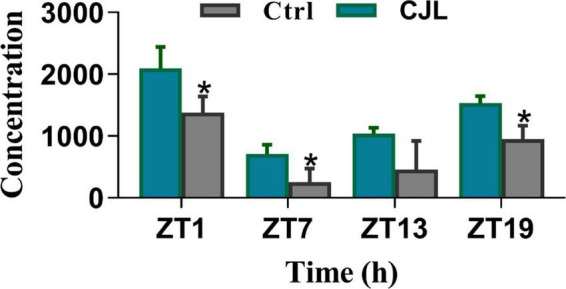
Chronic jetlag (CJL) increased corticosterone levels. This figure shows the plasma total corticosterone in C57/BL6 mice treated with phase advance CJL for 30 days, on four different time points (ZT1, ZT7, ZT13, and ZT19). Total corticosterone in a sample of tail blood was measured. All data are presented as means ± SEM, *P < 0.05, **P < 0.01, student’s *t*-test (*n* = 4).

The blood corticosterone levels alter in response to several neurological conditions. For instance, the corticosterone level increases in response to stress or depression (Webb et al., 2010; Chu et al., 2016). Therefore, we measured blood corticosterone levels in mice treated with CJL and found that the corticosterone levels were significantly increased under CJL (Figure 8).

### The impact of chronic jetlag on total sleep

We tested whether CJL leads to sleep deprivation using a piezoelectric sleep analysis system. However, we did not find significant differences in the total sleep of CJL-treated mice compared to control mice. We further analyzed the percent sleep in the light and dark phases, which revealed that sleep was significantly different in CJL-treated mice as compared to control mice (Gao et al., 2020; Supplementary Figure 3).

## Discussion

Dysregulated circadian rhythm alters physiology and behavior through hormonal disruption (Crispim et al., 2011) and induces neurological aberrations (Saksvik et al., 2011). In the current study, mice exposed to CJL had altered glucose uptake levels in the brain, indicating decreased neuronal function. In addition, the differentially expressed genes in the brain identified by RNA sequencing and qPCR were associated with neurological disorders. We observed the alteration in the expression of serotonin transporter genes, serotonin receptors, the levels of serotonin in PFC, and levels of corticosterone in the blood. Our investigations also revealed that total sleep remained unchanged. Shiftwork is a complex phenomenon that constitutes parameters including sleep quality, fatigue level, and types of sleep problems (Saksvik et al., 2011). Shift-workers are vulnerable to various diseases (Logan and McClung, 2019), including sleep behavior disorder, sleep-wake state dissociation disorders (Sehgal and Mignot, 2011), anxiety, and depression (Wyse et al., 2017). In the current study, we found that exposure of mice to CJL altered regulatory pathways and the expression of genes of several neurological disorders. It is known that jetlag induces obesity and increases weight (Wyse et al., 2017); we found that metabolic functions and insulin-related pathways were affected in the mice treated with CJL.

Neurological problems such as depression alter glucose utilization and reduce the brain’s activity (Chu et al., 2016), hence altered energy consumption can be calculated to determine the changes in neuronal activation and hypometabolism by measuring cerebral metabolic rates of glucose using fluoro-2-deoxyglucose (FDG)-PET (Coleman et al., 2017). Our results indicated that glucose uptake level was reduced in the brain, which might be one reasons for the alteration in the expression of neurological diseases associated genes. NAc and PFC have been found with reduced activity in patients with neurological disorders (Dietz et al., 2014; Carlson et al., 2015; Jenkins et al., 2018); the transcriptome profiles revealed that a number of neurological disorders associated pathways such as Parkinson’s disease and Turner’s syndrome were altered in CJL-treated mice. Depression-related genes, specifically glucocorticoids, were also found to be affected in both NAc and PFC. In addition to NAc and PFC, the striatum, hypothalamus, and hippocampus (Videbech and Ravnkilde, 2004; Frodl et al., 2010) are also associated with neurological disorders (Jenkins et al., 2018). For instance, the hippocampus is involved in episodic, declarative, contextual, and spatial learning and memory deficits which often accompany depression (Videbech and Ravnkilde, 2004). We hypothesized that the neurological disorders-related genes might be altered in these regions. Our study revealed that the expression levels of *Drd1*/*2*, *Fosb, Grik2*, and *Grin2A* were altered in NAc, PFC, hippocampus, hypothalamus, and striatum. We have considered all those genes as CJL affected genes or genes with altered expressions, which were either upregulated or downregulated at at-least one time point. These genes have been reported to play a key role in stress, depression, and other neurological conditions (Lemke et al., 2014; Ruffle, 2014; Liu et al., 2015). In general, the alterations in the expression levels of genes indicate that CJL can increase the risk of several important neurological diseases, such as schizophrenia and Huntington’s disease. Solute carriers-associated genes have been considered interesting players in neurological disorders (Vincent and Jacobson, 2014). The expression levels of a number of solute carrier-associated genes were found to be altered. These genes include *Slc13a*5, *Slc16a1*, *Slc29a1*, *Slc2a1*, *Slc2a3*, *Slc37a4*, and *Slc45a1*.

Serotonin receptors are associated with several disorders, including schizophrenia, bipolar disorder, seasonal affective disorder, depression, stress, anxiety, major depression disorder, Alzheimer, and attention deficit disorder (Lohoff et al., 2011). RNAseq analysis revealed that expression levels of serotonin transporters were altered in NAc and PFC, which are promising therapeutic targets associated with serotoninergic and dopaminergic (DA) transmission (Itzhacki et al., 2018). The alteration in mRNA levels of *Htr2a*, *Htr4*, *Htr7*, *Htr5b*, *Htr6*, and *Htr3* indicate that serotonin may increase the risk of CJL mediated neurological disorders. The raphe nucleus is an important region associated with 5-HT synthesis and release, whereas the dorsal raphe nucleus is implicated in depressive disorders (Xiao et al., 2017). Hence, the increased mRNA levels for serotonin transporter and tryptophan hydroxylase enzymes in the raphe nucleus further indicate that serotonin signaling is at least involved in CJL-mediated risk of neurological disorders. Tryptophan hydroxylase (*Tph1/2*) is a key enzyme in the 5-HT synthesis pathway (Vincent et al., 2018), and *Htr1A* and *Htr1B* are auto-receptors involved in regulating serotonin release (You et al., 2016).

Although the corticosterone level was increased in the blood, this increase was not uniform as expected. The difference in the hormonal level of control and CJL mouse was higher at ZT1 but much lower at ZT 13. Nonetheless, the results indicate that CJL can affect peripheral hormones. Moreover, Sleep is an important component of health (Halperin, 2014). Sleep analysis revealed that the overall sleep percentage remained unchanged in CJL-exposed mice compared to control mice, hence confirming that neurological conditions were not caused by sleep deprivation.

## Conclusion and future recommendation

In conclusion, these findings suggest a molecular relationship between CJL and neurological (such as depressive) disorders, which are induced by hormonal disruption. However, this study was limited by some factors such as testing different intensities of light, long term exposure of mice to CJL, and molecular profiling of remaining brain regions. Nevertheless, the results presented in this manuscript can further be implicated in investigating how CJL increases susceptibility to neurological diseases. Moreover, further studies should focus on investigating the specific markers and most relevant genes with neurological conditions, and behavioral changes in mice exposed to CJL like conditions.

## Data availability statement

The datasets presented in this study can be found in online repositories. The names of the repositories and accession numbers can be found below: Gene Expression Omnibus (GEO): GSE153540; Figshare: https://doi.org/10.6084/m9.figshare.c.5054273.

## Ethics statement

All animal experiments were approved by the Institutional Animal Use and Care Committee at Tongji Medical College, Huazhong University of Science & Technology. All experiment methods were performed in accordance with the relevant guidelines and regulations.

## Author contributions

RS and SK performed all the experiments, analyzed the data, and wrote and revised the manuscript. FA revised and updated the revised manuscript. GN edited and reviewed the revised manuscript. MX supervised the overall work. All authors contributed to the article and approved the submitted version.

## Footnotes

1
http://identifiers.org/geo:GSE153540

2
https://doi.org/10.6084/m9.figshare.c.5054273
