# Supplementary material for: Chronic jet lag-like conditions dysregulate molecular profiles of neurological disorders in nucleus accumbens and prefrontal cortex
Source: Front Neuroinform. 2022 Dec 13;16:1031448. doi: 10.3389/fninf.2022.1031448 (PMC9792783; doi:10.3389/fninf.2022.1031448)
Supplement: Supplementary file 1 [file Data_Sheet_1.docx]

**Supplementary materials**

Table S1: List of primers

**Table 2.2 Cont.**

| **GENE** | **FORWARD PRIMER SEQUENCE** | **REVERSE PRIMER SEQUENCE** |
| --- | --- | --- |
| *Arntl* | 5’AAGGTGGCCCAAAGAGGACT 3' | 5'GCCTCCTGGAGAAGAGGCAT 3' |
| *Drd-1* | 5'ACCTAAGCCACTGGAGAAGC 3' | 5'AACAGCAAGCCCTAGGGAAC 3' |
| *Drd-2* | 5'CCCACCACGGTCTACATAGC 3' | 5'CCATTGGGCATGGTCTGGAT 3' |
| *Fos-b* | 5'ACATGCCAGGAACCAGCTAC 3' | 5'GGGTAAGTGTCTCTTCTCGGG 3' |
| *Grik-2* | 5'ACTCCCACTGAGGTTTAGTCC 3' | 5'GTTTTCCACCACGTCTGAGTC 3' |
| *Grin-2a* | 5'TGACAAGAAGTTCCAGAGACCT 3' | 5'ATGTCTACGCCCGCTTTCG 3' |
| *Grin-2b* | 5'CGGCATAGGCAGACCTTTTC 3' | 5'TCCCCTTCCAGAGGAATTGA 3' |
| *Hdac1* | 5'ACCATCAAAGGACACGCCAA 3' | 5'AGTCGTTGTAGGGCAGCTCA 3' |
| *Hdac9* | 5'GGTCCAATCAGATGCTGCGA3’ | 5'GCCATGGAACTGTATGCCAGA 3' |
| *Htr-2a* | 5'TCTCACCATTGCGGGAAACA 3' | 5'GCCACCGGTACCCATACAG 3' |
| *Jarid2* | 5'TGCTGTGCCTGGAGTGACTA 3' | 5'GCAGCAGAGTGTGGACTACG 3' |
| *Sin3a* | 5'GTCAGCATACCAGCGGAAGG 3' | 5'GTCTGACCAACGCTCTGCTT 3' |
| *Sirt-2* | 5'AGCCGGACCGATTCAGACT 3' | 5'TTCGAGGGTCAGCTCGTCTA 3' |
| *Slc-13a5* | 5'GACTCCAAGCAGGCTGATGC 3' | 5'TGGCCTCCACAATGGGTATC 3' |
| *Slc-16a1* | 5'TCATGACCCTTTCACTCACC 3' | 5'CCAATCTGGTCCATCTTTCC 3' |
| *Slc-29a1* | 5'CGCTTACTCCAAGCACAAGAG 3' | 5'ACATGGAACAGGCACAGAAG 3' |
| *Slc-2a1* | 5'CGGTAGCTGCCACCCACTTC 3' | 5'CTAGAACCTGGCAGCCCAGC 3' |
| *Slc-2a3* | 5'TTGGGTTCCCTGTCCCTGTT 3' | 5'TTGCGCCTCCTAGCTGTAGT 3' |
| *Slc-37a4* | 5'AGGGCAAAAAGGGCTCATCA 3' | 5'CACCCAGAGATAGGGGGACA 3' |
| *Slc-45a1* | 5'ACTGTCTCTGCGGCATCTCT 3' | 5'GTCTCCCTGGAACACCACCT 3' |
| *Smarca1* | 5'GCAGACCGCGCAAAGAGATT 3' | 5'TGGCGATAGTCTCCAACGGA 3' |
| *Smarcad1* | 5'AAGCCTTTCTCCTGCTCTGG 3' | 5'AAGCGGGAGAGGAAGCTGAT 3' |
| *5htt* | 5'CAGCGACGTGAAGGAAATGC 3' | 5'AGAAACAGAGGGCTGATGGC 3' |
| *5ht1a* | 5'TACGTGAACAAGAGGACGCC 3' | 5'AAAGCGCCGAAAGTGGAGTA 3' |
| *5ht1b* | 5'ACCCCGGCTAACTACCTGAT 3' | 5'CTAGTGTCCAGCGTCCAGTG 3' |
| *Bdnf* | 5'AGCTCCGGGTTGGTATACTG 3' | 5'GCCTTCATGCAACCGAAGTA 3' |
| *Fos-b* | 5'ATGGTGAAGACCGTGTCAGG 3' | 5'GTTGATCTGTCTCCGCTTGGA 3' |
| *Tph1* | 5'TGCGAAGGAAGACGTTATGGA 3' | 5'ATTCGCACTGCGTGACTTTG 3' |
| *Tph2* | 5'TGAGAGCATTTGGACGGAGG 3' | 5'TTAAATCCTGGATGGTCGGCA 3' |
| *Cry1* | 5'ATCCACCATTTAGCCAGACACG3' | 5'GACAGCCACATCCAACTTCCAG 3' |
| *Cry2* | 5'GGGGACTCTGTCTATTGGCATCT 3' | 5'CTGGCTCTTGGGTAGGCATCT 3' |
| *Per1* | 5' TTGGCAGGCTTCGTGGACTTG 3' | 5' GCGGGAACGCTTTGCTTTAGAT 3' |
| *Per2* | 5' GACAGCAGCTTCTGGTCTGGACT 3' | 5' TTCTGAGTGTCTGAGGGCTCGTT 3' |
| *Per3* | 5'TCGCCCTACGGTTGCTATCTTC 3' | 5'TTCGTTTGTGCTTCTGCCTCTC 3' |
| *Dec1* | 5'ACCTGTCAGGGATGGATTTTGC3' | 5'CTTGCTGTCTTCGCTCCGTTTT 3' |
| *Dec2* | 5'CCAAGGATACCTACAAGTTACCG 3' | 5'ACTACTGCTTTCTCCAAATGCCC 3' |
| *Clock* | 5'CCTATCCTACCTTCGCCACACA3' | 5'TCCCGTGGAGCAACCTAGAT 3' |
| *Nr1d1* | 5'ACGGCAAGGCAACACCAAGA3' | 5'CGCACCATCAGCACCTCAAA 3' |
| *Rorb* | TAGCTCCCGGGATAACAATG 3' | 5'GCCAGCTGATGGAGTTCTTC 3' |
| *Rora* | 5' GGCTCGCTAGAGGTGGTGTTT 3' | 5' CGATTTCGTCTTCGGTCAGGT 3' |
| *Rory* | 5'TGCAAGACTCATCGACAAGG 3' | 5'AGGGGATTCAACATCAGTGC 3' |
| *Dbp* | 5'TGAGACTTTTGACCCTCGGA 3' | 5'CCTCTTGGCTGCTTCATTGT 3' |


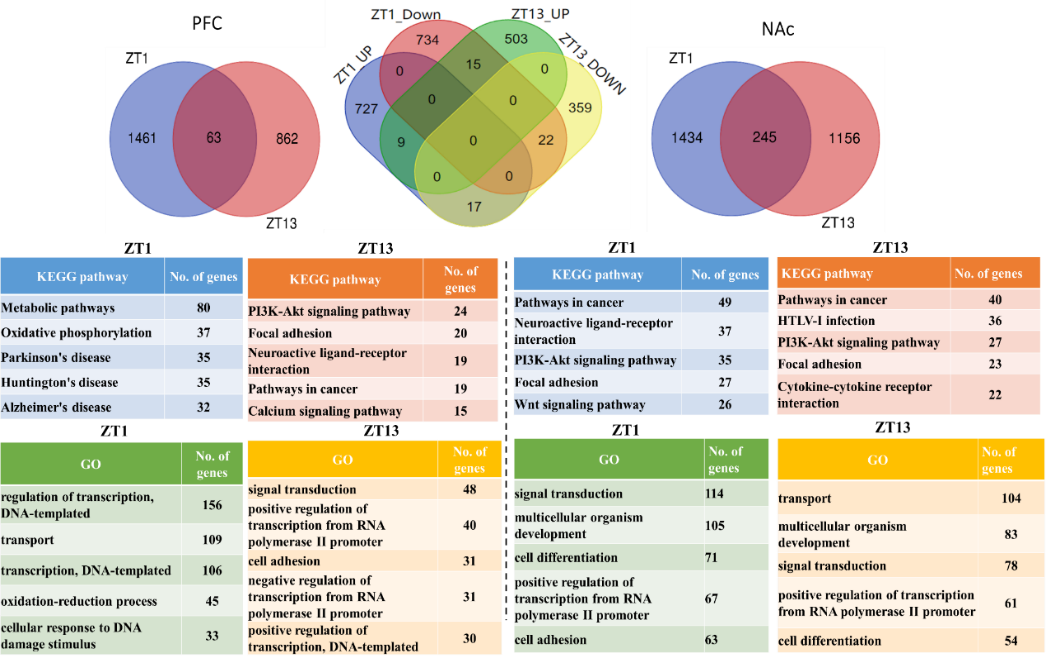


Figure S1. The statistical enrichment analyses of KEGG and GO terms for mouse brain (PFC and NAc) genes against the entire mouse proteome, using the hypergeometric test. Altogether, significantly overrepresented KEGG pathways were detected (p-value < 0.001). For GO terms, the top most significantly enriched biological processes (p-value < 0.001) were shown. The results were visualized using an online tool (DAVID) [1].


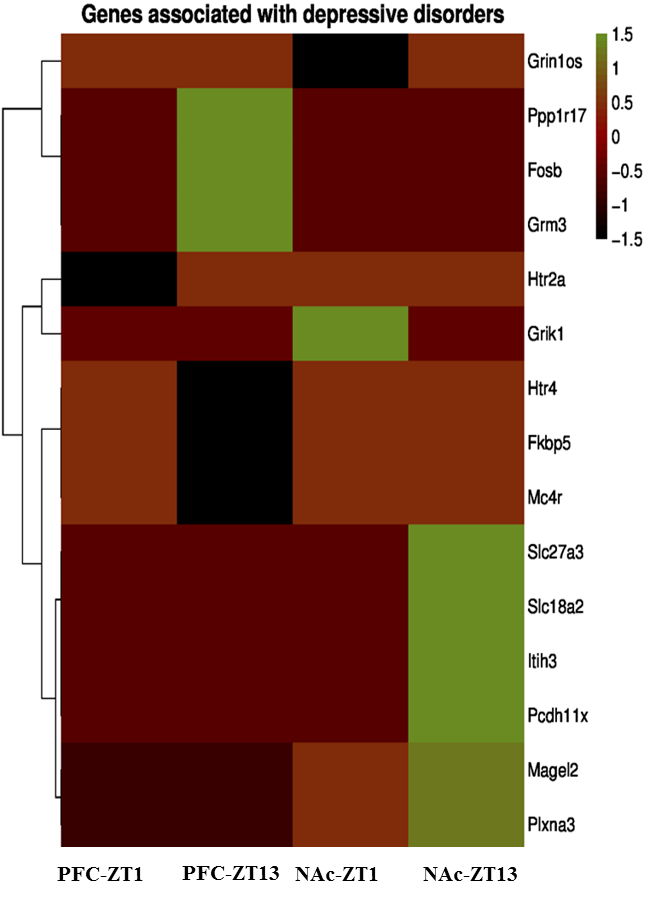


**Figure S2.** Differentially expressed genes that are associated with depressive disorders. Only those genes were selected which were found differentially regulated in both PFC and NAc.


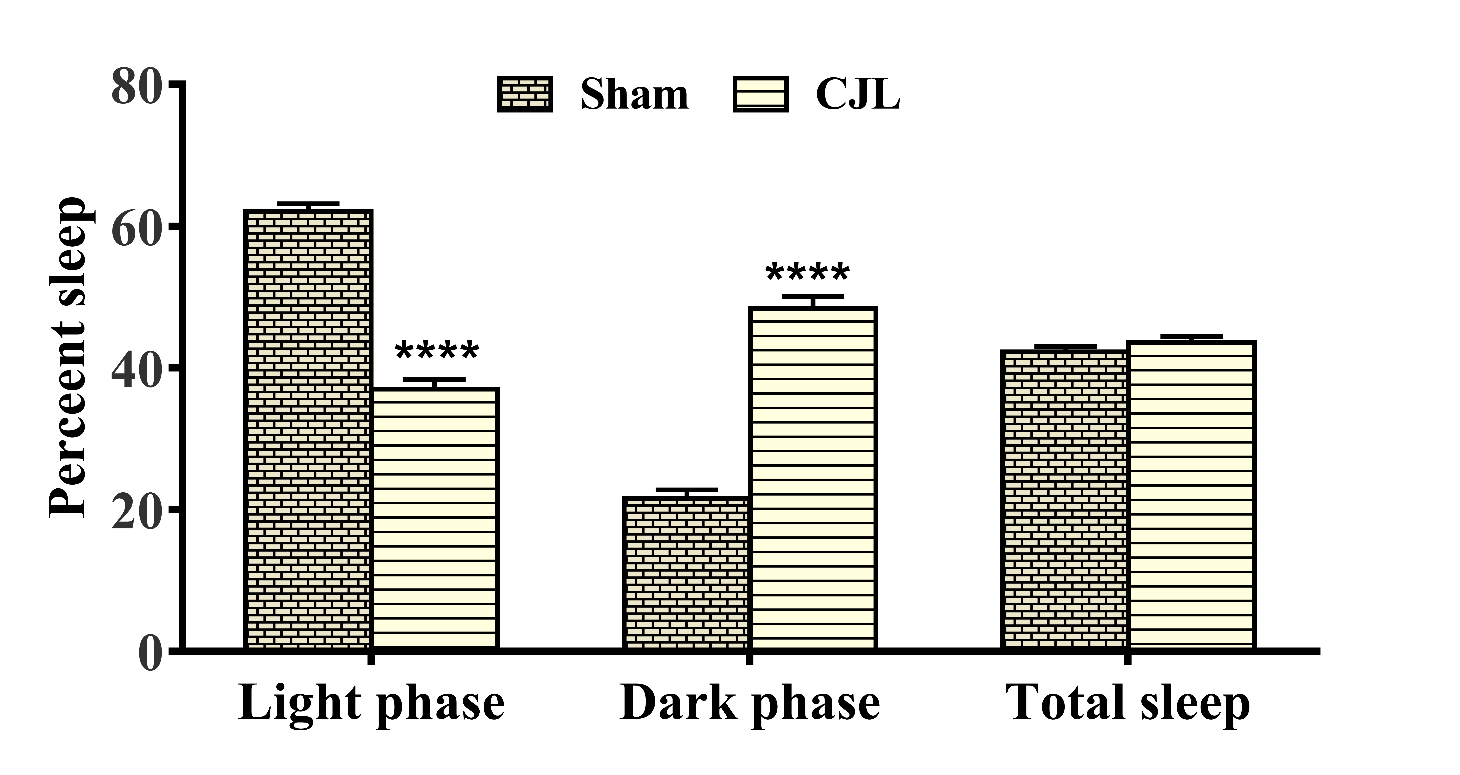


**Figure S3.** Sleep analysis using Piezo sleep system. Total sleep remained unaltered on CJL treatment. P value (two-way ANOVA) for both light and dark phase is < 0.0001 and for total sleep is 0.8400. All data are presented as means ± SEM, ****P < 0.0001 [1].

**
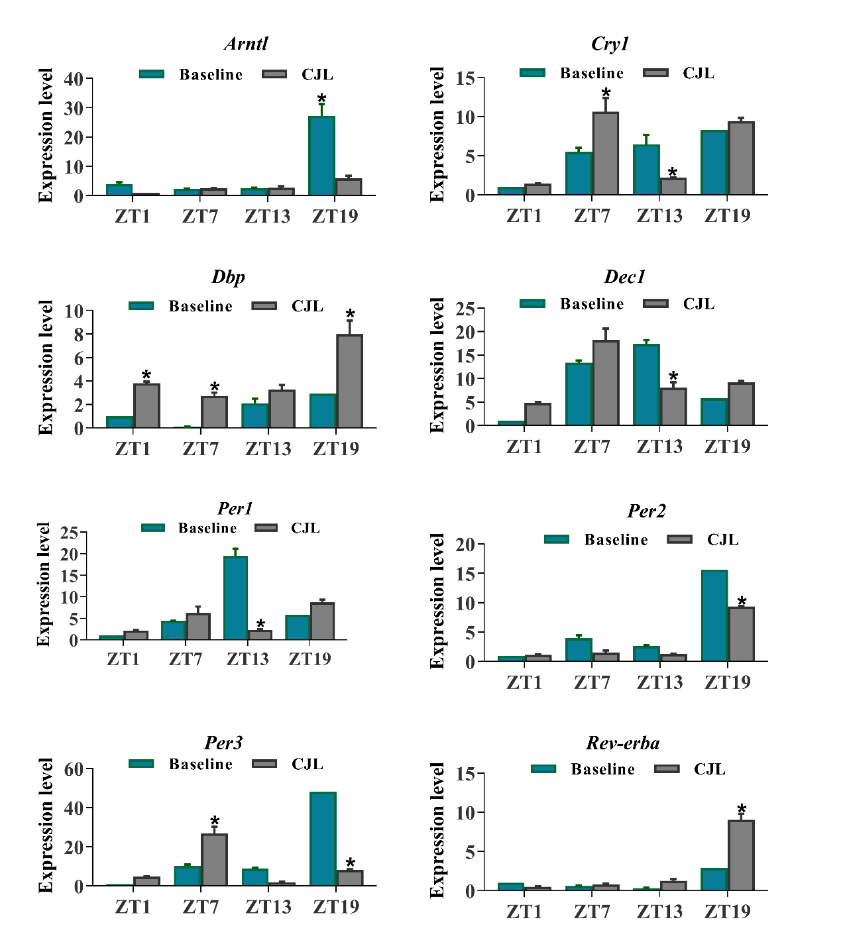
**

**Figure S4.** CJL altered the mRNA levels of clock genes in the hypothalamus. This figure shows mRNA levels in hypothalamus extracts from baseline and CJL treated animals (n = 3 per group), as assayed by three independent qPCR assays, at 4 different time points (ZT1, ZT7, ZT13, and ZT19). Results are expressed as mean ± SEM. **P* < 0.01, 1two-way ANOVA.


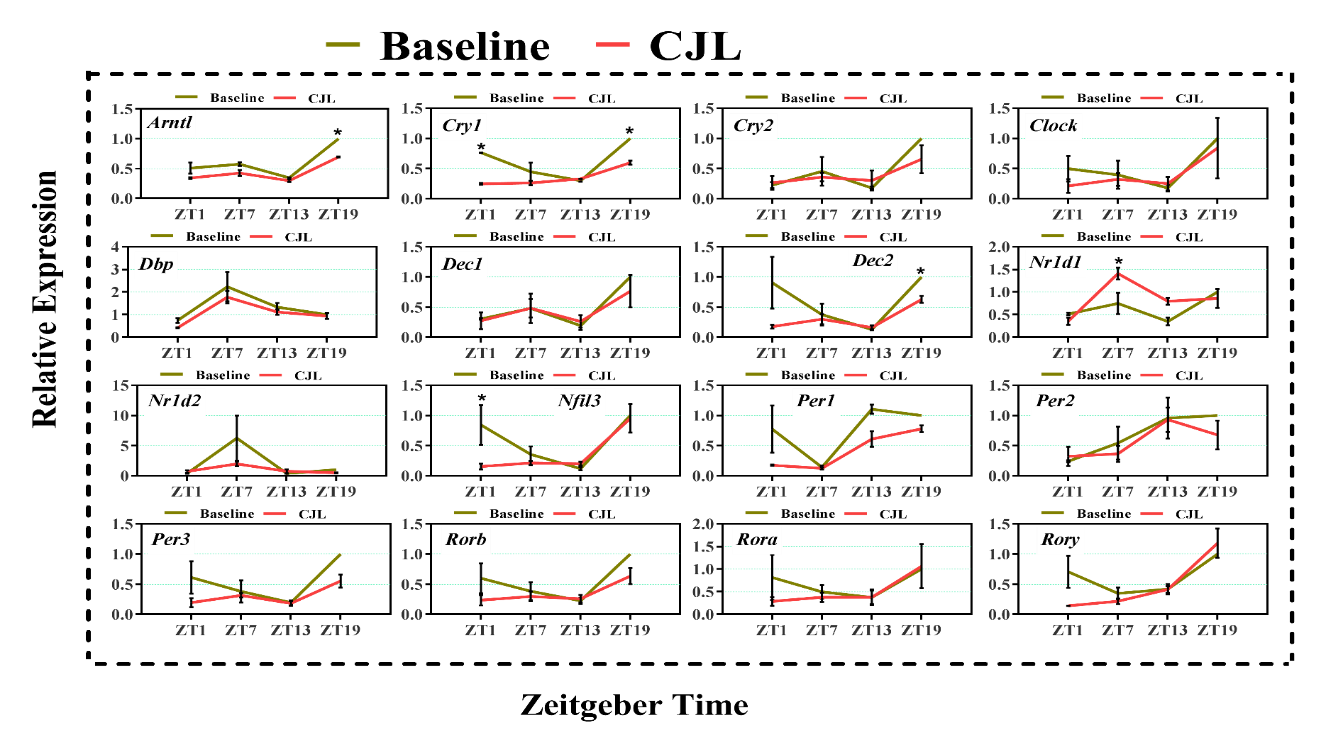


**Figure S5.** CJL altered the mRNA levels of clock genes in the raphe nucleus. This figure shows mRNA levels in raphe nucleus extracts from baseline and CJL treated animals (n = 3 per group), as assayed by three independent qPCR assays, at 4 different time points (ZT1, ZT7, ZT13, and ZT19). Results are expressed as mean ± SEM. **P* < 0.01, 1two-way ANOVA.

**
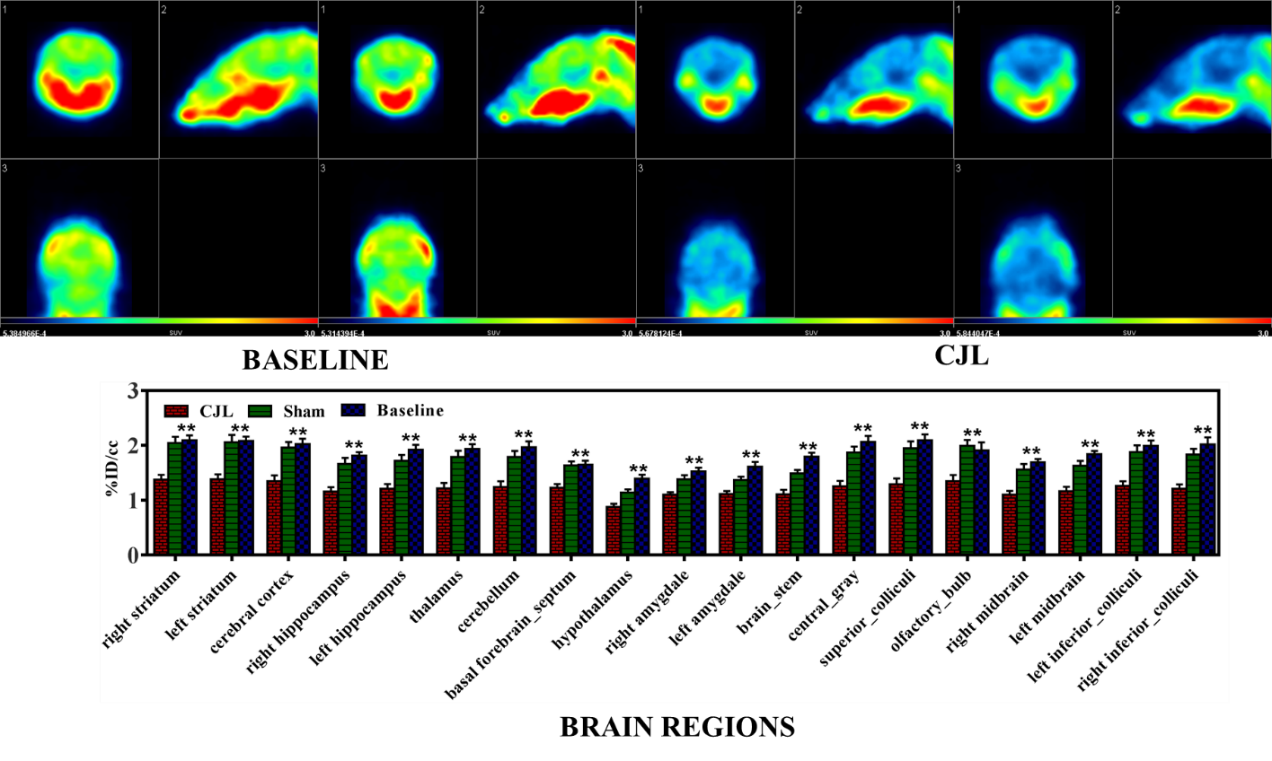
**

**Figure S6.** Alternations of glucose metabolism by small animal PET scanning. Representatives of the glucose uptake of CJL and Baseline group. The changes of glucose uptake in brain regions of CJL group. Glucose uptake of brain regions including striatum, cortex, hippocampus, thalamus, cerebellum and brain stem is significantly decreased in CJL mice. Data are presented as means ± SEM, **P < 0.01; ***P < 0.001 [1].

References

1. Gao Q, Khan S, Zhang L. Brain activity and transcriptional profiling in mice under chronic jet lag. Sci data. 2020;7:361.
